# E3 ubiquitin ligases in neurodegenerative diseases

**DOI:** 10.1016/j.mmr.2026.100032

**Published:** 2026-04-27

**Authors:** Duan Lu Hou, Josephine Ho, Tuchen Guan, Xiao-Xue Dong, Li Zeng, Laurie H Sanders, Yun-Cheng Wu, Eng King Tan, Zhi Dong Zhou

**Affiliations:** a National Neuroscience Institute, Singapore 308433, Singapore; b Department of Neurology, Shanghai General Hospital, Shanghai Jiao Tong University School of Medicine, Shanghai 200080, China; c Nanyang Technological University, Singapore 639798, Singapore; d Departments of Neurology and Pathology, Duke University School of Medicine, Durham, NC 27710, USA; e Duke Center for Neurodegeneration and Neurotherapeutics, Duke University, Durham, NC 27710, USA; ^f^Department of Neurology, Singapore General Hospital, Singapore 169608, Singapore

**Keywords:** Amyotrophic lateral sclerosis, Alzheimer’s disease, Huntington’s disease, Multiple system atrophy, Neurodegenerative diseases (NDs), Parkinson’s disease, Ubiquitin-proteasome system (UPS), E3 ubiquitin ligase

## Abstract

Neurodegenerative diseases (NDs) are characterized by progressive neuronal loss and proteostatic failure, driven by impaired clearance of misfolded proteins via the ubiquitin-proteasome system (UPS) and autophagy. In UPS, E3 ubiquitin ligases are crucial for regulating protein ubiquitination and degradation. Mutations in E3 ligases, along with dysfunctions of specific ligases such as Parkin, the C-terminus of HSC70-interacting protein (CHIP), and tripartite motif-containing proteins, have been identified as key factors in the buildup of amyloid-β, α-synuclein, tau, trans-active response DNA-binding protein 43, and mutant huntingtin. These accumulations are associated with NDs like Parkinson’s disease, Alzheimer’s disease, Huntington’s disease, and amyotrophic lateral sclerosis. Therapeutic strategies targeting E3 ligases, particularly proteolysis-targeting chimeras (PROTACs), are being developed for ND treatment and are currently in clinical trials. These approaches aim to enhance E3 ligase activity and promote selective protein degradation. Here, we examine how individual E3 ligases influence cell-fate decisions in NDs, showing that their substrate selection determines whether neurons survive or die. Building on this knowledge, we present an innovative therapeutic pipeline that includes ligase activators, PROTAC degraders, and miRNA switches, which are molecules designed to transition from research to clinical application.

## 1 Background

Neurodegenerative diseases (NDs) represent an escalating global health crisis, characterized by progressive neuronal loss and the pathological accumulation of misfolded protein aggregates that exhibit prion-like propagation [1], [2]. This progressive neuronal degeneration is driven proteinopathies, disrupted RNA homeostasis, glial dysfunction, neuroinflammation, and failures in lysosomal and autophagic processes [3], and it remains incurable today [4]. Emerging therapies for NDs, especially age-related NDs, include viral vector-based gene therapy [5], immunotherapy targeting pathological proteins [6], stem cell-derived replacement [7], and mesenchymal stem cell-derived extracellular vesicles with immunomodulatory properties [7], [8].

Despite clinical heterogeneity spanning Parkinson’s disease (PD), Alzheimer’s disease (AD), Huntington’s disease (HD), amyotrophic lateral sclerosis (ALS), and rarer disorders such as multiple system atrophy (MSA) and dentatorubral-pallidoluysian atrophy (DRPLA), these conditions share a unifying pathological hallmark: the failure of cellular proteostasis networks to clear toxic protein species such as amyloid-β (Aβ), α-synuclein (α-syn), tau, trans-activation response (TAR) DNA-binding protein 43 (TDP-43) and huntingtin (Htt) [9]. A central mechanism underlying this collapse is dysfunction of the ubiquitin-proteasome system (UPS), as evidenced by the presence of ubiquitin (Ub) within disease-defining inclusions and by genetic linkage of multiple E3 Ub ligases to familial NDs [4], [10]. E3 ligases serve as gatekeepers of proteostasis, directing misfolded proteins toward either proteasomal degradation or sequestration into aggregates, a fate complicated by the aggregation paradox, wherein initial aggregation may be cytoprotective while mature inclusions drive neurodegeneration [10], [11], [12]. This unique position makes E3 ligases promising therapeutic targets, as their modulation could reduce toxic oligomers while preserving beneficial inclusion bodies [13], [14].

Despite increasing evidence of the importance of E3 ligases, the development of therapies remains limited because of a disease-focused view that hides common mechanisms. This review systematically integrates current knowledge of E3 ligases in neurodegeneration. We elucidate their mechanistic contributions to ND pathogenesis across diverse cellular pathways and highlight the therapeutic potential of targeting proteostatic failure, the core pathology shared by all NDs, rather than merely addressing disease-specific symptoms.

## 2 Ubiquitination

**Fig. 1:**
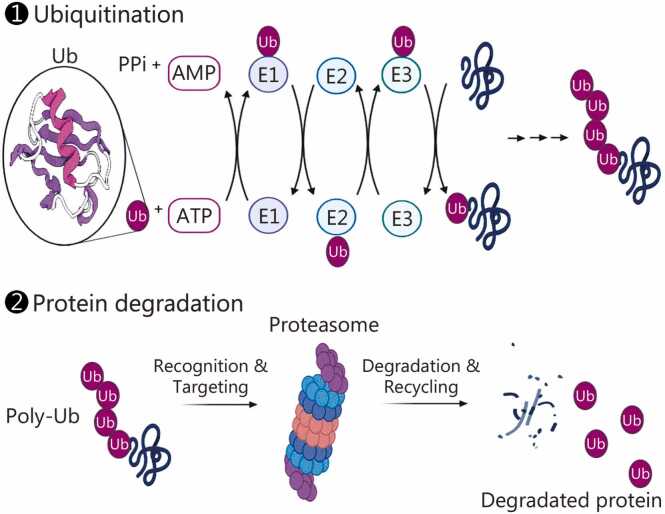
Ubiquitination and related protein degradation. ATP-dependent E1 first activates Ub (C-terminal Gly76) and forms an E1-Ub thioester. Ub is then transferred to the catalytic Cys of the E2 conjugating enzyme. Finally, an E3 ligase catalyzes the attachment of Ub to a substrate lysine (or N-terminus), forming an isopeptide bond (or peptide bond) and regenerating free E2. Proteasomal degradation (K48 chain-related) of the substrate will cleave it and release poly- or mono-Ub. PPi. Pyrophosphatic acid; E3. E3 ubiquitin ligase; Ub. Ubiquitin; E1. Ubiquitin-activating enzyme; E2. Ubiquitin-conjugating enzyme; ATP. Adenosine triphosphate; AMP. Adenosine monophosphate.

Ubiquitination, also known as ubiquitylation, is a crucial post-translational modification of proteins. It plays an essential role in proteasome-mediated protein degradation, DNA repair, and autophagy during development and disease [15]. The process involves attaching Ub, a highly conserved 76-amino-acid protein, to lysine residues or, less frequently, to the N-terminus of target proteins. The ubiquitination pathway is regulated by three main enzymes: E1 (the Ub-activating enzyme), E2 (the Ub-conjugating enzyme), and E3 (the Ub ligase) [16]. E1 activates Ub by forming a thioester bond with its C-terminus, then transfers it to E2. E3 ligase facilitates the transfer of Ub from E2 to the lysine residue on the target protein. The primary function of ubiquitination is to tag short-lived, misfolded, or damaged proteins for destruction by the 26S proteasome, which degrades 70%–80% of cellular proteins [17]. This proteolytic complex consists of a 19S regulatory particle and a 20S core particle; upon substrate recognition, it releases peptide fragments and recycles Ub molecules [18]. Ub has seven lysine sites (K6, K11, K27, K29, K33, K48, K63) and an N-terminal methionine (Met1), each forming specific linkages that determine downstream functional outcomes. Proteins targeted for degradation are typically marked with K48-linked Ub chains, the most common signal for proteasomal degradation, and occasionally with K11-linked chains [19], [20]. Beyond proteolysis, distinct Ub chain topologies regulate diverse cellular processes (Fig. 1**;** Table 1) [19], [21], [22], [23], [24], [25], [26]. K11 linkages additionally mediate cell cycle control and membrane trafficking [20], while K63 chains regulate protein trafficking, signaling, immune responses, and DNA repair [27].

**Table 1:** Functional sites of Ub.

**Ub sites**	**Key functions**	**References**
N-terminal Met1	NF-κB and IFN signalling	[19], [21]
K6	DNA repair; Mitochondrial homeostasis	[22]
K11	Cell cycle regulation; 26S proteasome-mediated protein degradation; Membrane trafficking; Innate immune response	[19], [23]
K27	Innate immune response activation; Antiviral signalling; DNA repair; Mitophagy	[19], [24]
K29	Innate immune response; Selective autophagy; Proteasomal degradation; AMPK-related protein kinase regulation	[19], [24]
K33	Innate immune response	[19], [24]
K48	26S proteasome-mediated degradation	[19], [24], [25]
K63	DNA repair; Cytokine signalling; Autophagic degradation; Immune response regulation	[19], [24], [26]

Ub. Ubiquitin; NF-κB. Nuclear factor κB; IFN. Interferon

**Fig. 2:**
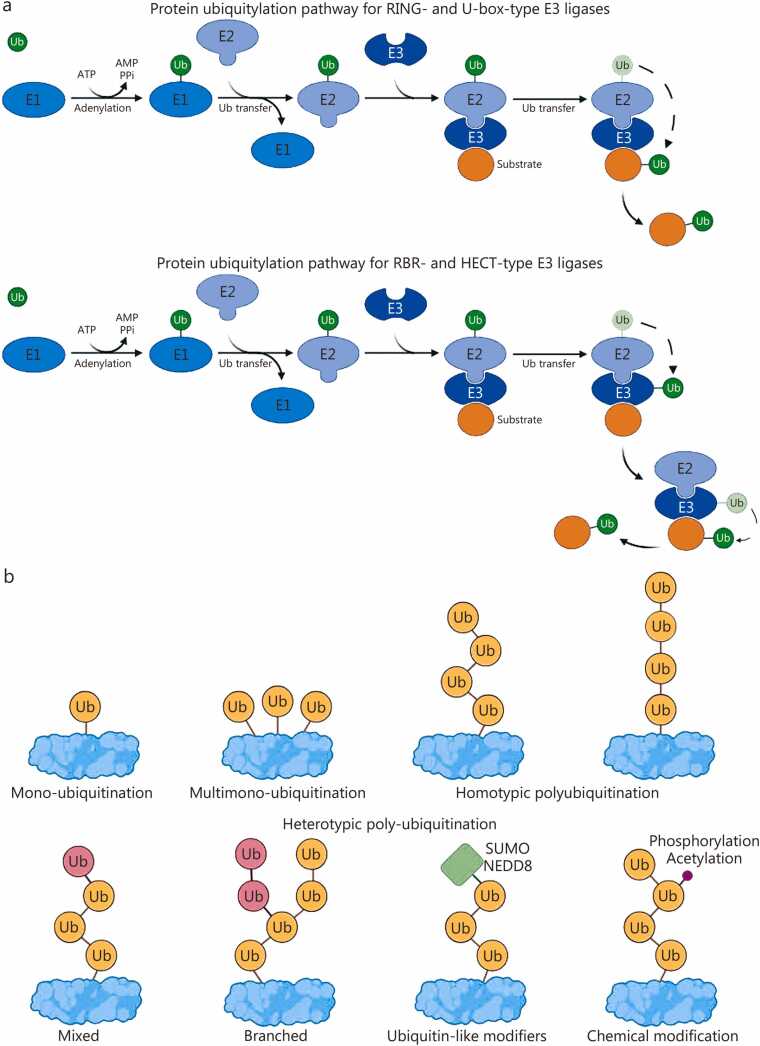
E3 ligase classification and ubiquitin (Ub)-chain topologies. This figure provides a detailed overview of E3 ubiquitin ligases and the various ubiquitin chain structures they produce. **a** The protein ubiquitylation pathways for different types of E3 ligases: RING, U-box, RBR, and HECT. RING- and U-box-type E3s function as scaffolds that dock the E2-Ub thioester and facilitate direct transfer of Ub to the substrate lysine. In contrast, RBR- and HECT-type E3 ligases employ a two-step mechanism: Ub is first transferred from E2 to the catalytic cysteine of RING2 (in RBR) or the C-terminal lobe (in HECT), and then to the substrate. **b** It summarizes chain topologies, including mono-, multimono-, homotypic (uniform linkage), heterotypic (mixed linkages), and branched chains, which illustrate how identical Ub units can encode distinct cellular fates. It also separates polyubiquitination into homotypic and heterotypic forms based on linkage types and chain structures, including mixed, branched, and NEDD8- or phosphorylation-modified forms. RING. Really interesting new gene; RBR. RING-between-RING; HECT. Homologous to the E6-AP carboxyl terminus; SUMO. Small ubiquitin-related modifier; NEDD8. Neural precursor cell expressed developmentally downregulated protein 8; ATP. Adenosine triphosphate; AMP. Adenosine monophosphate; PPi. Pyrophosphatic acid.

Ubiquitination is classified based on the number of Ub units attached to a substrate (Fig. 2). Mono-ubiquitination involves a single Ub molecule linked to one lysine residue. Multimono-ubiquitination refers to one Ub attached to multiple lysines. Polyubiquitination involves the formation of Ub chains on substrate proteins via isopeptide bonds (lysine residues) or peptide bonds (the N-terminus). These chains are primarily categorized by two factors: first, by linkage type, which distinguishes homotypic chains with uniform linkages (e.g., K48- or M1-linked chains) from heterotypic chains with mixed linkages; and second, by topology, which differentiates unbranched from branched structures [25], [28]. Homogeneous polyubiquitin chains contain Ub molecules linked at the same lysine residue. In contrast, heterogeneous chains, which are less common, have Ub linked at different lysines. Ub forms linear polyubiquitin chains connected through its N-terminal Met1, while branched chains feature at least one Ub with multiple lysine acceptor sites [29].

Ubiquitination has diverse non-degradative roles, including regulating protein-protein interactions, signal transduction, gene expression [30], kinase activity [31], cellular localization, and the DNA damage response by recruiting repair factors [32]. It also affects endocytosis, chromatin remodeling, immune signaling, and lipid metabolism [33], [34], [35]. E3 Ub ligases facilitate these processes through specialized mechanisms [36]. For instance, a Myc-binding really interesting new gene (RING)-type E3 ligase specifically catalyzes esterification of threonine residues (rather than serine) [37], while Cullin-RING ligases recognize C-terminal degrons to shape the eukaryotic proteome [38]. In cancer, synoviolin 1 (SYVN1), a RING-type E3 ligase, promotes hepatocellular carcinoma metastasis by modulating heat shock protein 90 (HSP90)-mediated ubiquitination of eukaryotic elongation factor 2 kinase [39]. Dysregulation of ubiquitination is involved in various diseases, including cancer, metabolic disorders, and neurodegenerative conditions [40]. Targeting the UPS has become a promising therapeutic approach, with proteasome inhibitors already used clinically for certain cancers [41].

## 3 E3 Ub ligases

E3 Ub ligases have multiple substrates, and vice versa, which poses challenges for therapeutic specificity. E3 ligases differ in structure and function, with low sequence homology and compositional differences across families [19]. The four main subfamilies, RING finger, U-box, RING-between-RING (RBR), and homologous to the E6-associated protein (E6AP) C-terminus (HECT), facilitate Ub transfer from E2 enzymes to target proteins through different mechanisms (Fig. 2). Specifically, RBR and HECT ligases catalyze direct transfer, while RING and U-box ligases act as scaffolds for indirect transfer (Fig. 2) [42], [43].

### 3.1 RING-finger E3 ligases

RING-finger E3 ligases constitute the most prominent E3 family, with over 600 members expressed in humans [44]. Unlike HECT E3 ligases, RING E3 ligases mediate direct transfer of Ub from the E2 enzyme to the substrate without forming a covalent E3-Ub intermediate [45]. The RING finger domain, defined by a regular pattern of cysteine and histidine residues that coordinate Zn²⁺, stabilizes the E2-Ub thioester in a closed conformation for nucleophilic attack [45], [46]. This arrangement is reinforced by Ub-RING contacts and an arginine residue bridging E2 and Ub [46]. These ligases can function as monomers, dimers, or multi-subunit complexes and are categorized into 4 main subfamilies: membrane-associated RING-CH (MARCH), protease-associated transmembrane RING (PA-TM-RING), tripartite motif (TRIM), and RING-Ub interacting motif (RING-UIM) [44].

Subfamily-specific functions reveal distinct disease links: Skp1-Cullin1-F-box (SCF) E3 ligase dysfunction promotes polyQ aggregation in HD, as evidenced by reduced Cullin1 and Skp1 levels in HD mice [47]; MARCH proteins contain GXXXG motifs in their transmembrane helices that mediate oligomerization and substrate recognition, playing roles in MHC-II antigen presentation, viral immune evasion, and mitochondrial quality control in NDs [48]; PA-TM-RING proteins such as ring finger protein 13 (RNF13) are upregulated in neurons extending neurites on fibronectin, implicating this subfamily in vesicular trafficking and signaling [49]; and TRIM proteins regulate protein aggregation in NDs, with TRIM11 clearing α-syn aggregates in PD and TRIM21 clearing tau in AD, while TRIM18-mediated degradation of protein phosphatase 2 A (PP2A) drives tau hyperphosphorylation and neurodegeneration [50].

### 3.2 U-box E3 ligases

U-box E3 ligases constitute a small family specialized in post-translational protein quality control. In humans, the U-box domain (a 70-amino-acid C-terminal motif structurally resembling the RING finger domain) mediates direct transfer of Ub from E2 to substrates without forming an E3-Ub intermediate [19]. CHIP [heat shock cognate 71 kD protein (HSC)-interacting protein] is a dimeric U-box E3 ligase implicated in NDs, including PD [51]. Functioning as both a co-chaperone and E3 ligase, CHIP collaborates with Bcl-2-associated athanogene 1 (BAG-1) to regulate the HSC/ HSP70 chaperone system, directing irreparably misfolded proteins toward either refolding or UPS-mediated degradation [51]. Through its U-box domain, CHIP ubiquitinates α-syn and facilitates its degradation via both the UPS and lysosomal pathways [52]. Notably, CHIP overexpression reduces α-syn oligomerization through dual mechanisms: 1) HSP70-dependent pathway. CHIP’s TPR domain recruits HSP70-α-syn complexes, priming them for proteasomal degradation; and 2) HSP70-independent pathway. CHIP’s U-box domain directly binds α-syn hydrophobic regions and catalyzes K63-linked ubiquitination, marking oligomers for lysosomal clearance. This bifurcated action enables CHIP to target both soluble and aggregated forms of α-syn [52].

### 3.3 RBR E3 ligases

RBR E3 ligases are newly discovered hybrids of HECT and RING E3 ligases, containing two RING finger domains (RING1 and RING2), and an in-between-RINGs (IBR) zinc-binding domain situated between the two RINGs [53]. Similar to HECT E3 ligases, RBR E3 ligases catalyze ubiquitination through the formation of an E3-Ub intermediate. The E2-Ub complex is recruited to RING1, while RING2 contains the active-site cysteine for ubiquitination [46].

Parkin is a 465-amino-acid RBR E3 ligase involved in PD pathogenesis [54]. Its activity is tightly controlled through intramolecular interactions among its domains: RING0, RING1, RING2, and the repressor element protein (REP), where RING0 blocks the catalytic site in RING2 while REP binds RING1 to prevent E2 access [54], [55], [56]. This autoinhibitory conformation keeps Parkin inactive until activated by phosphatase and tensin homolog (PTEN)-induced kinase 1 (PINK1) [54], [55], [56]. When mitochondrial damage and depolarization occur, PINK1 accumulates on the outer mitochondrial membrane and recruits cytosolic Parkin, triggering mitophagy, the selective autophagic removal of dysfunctional mitochondria [57]. Parkin also influences nuclear factor κB (NF-κB) and mitogen-activated protein kinase (MAPK) pathways through interactions with receptor-interacting protein kinase 1 (RIPK1) and other substrates [54], [55], [56]. Therapeutic approaches aimed at restoring Parkin-mediated mitophagy in PD focus on activating PINK1. *In vitro* studies show that PINK1 autophosphorylation at Ser402 is specifically required for Parkin recruitment, whereas Ser228 autophosphorylation enhances substrate phosphorylation but is not essential for Parkin binding [54], [55], [56]. Small-molecule PINK1 activators that promote dimerization or allosteric binding to enhance autophosphorylation are promising therapeutic options [58].

### 3.4 HECT E3 ligases

HECT E3 ligases contain an approximately 350-amino-acid C-terminal HECT domain and an N-terminal substrate-binding region. The HECT domain comprises an N-lobe that recruits E2 and a C-lobe with a catalytic cysteine that forms a thioester intermediate with Ub. These lobes are linked by a flexible hinge, allowing the catalytic site to approach the E2-bound N-lobe for Ub transfer. When E3 binds, the E2-Ub complex adopts an open conformation, with Ub stabilized by the C-lobe [59]. After Ub transfers to the E3 cysteine, the C-lobe of Ub rotates approximately 130° away from E2 and moves nearer to the substrate [46]. The thioester bond of C-lobe-Ub is then positioned to the substrate’s lysine receptor, enabling nucleophilic attack by the substrate. The C-terminal region of HECT E3 influences substrate polyubiquitination; however, the mechanisms underlying its recognition and preference for specific linkage types remain unclear [46].

There are about 30 HECT E3 ligases in the human genome involved in many biological processes, including protein trafficking, immune responses, DNA damage repair, signaling, and apoptosis [60]. HECT E3 ligases can be divided into three groups based on their N-terminal domains: the neural precursor cell expressed developmentally down-regulated protein 4 (Nedd4) family, the HECT and regulator of chromosome condensation 1 (RCC1)-like domain (RLD) containing (HERC) family, and other HECTs [19].

#### 3.4.1 Nedd4

The Nedd4 subfamily is defined by 2–4 WW domains and an N-terminal C2 (Ca^2+^/lipid-binding) domain [20]. Its nine members include Nedd4-1, Nedd4-2, itchy E3 ligase [ITCH, also known as itchy/atrophin-1 interacting protein 4 (AIP4)], WW domain-containing E3 ligase 1–2 (WWP1–2), SMAD-specific E3 ligase 1–2 (Smurf1–2), and Nedd4-like E3 ligase 1–2 (NEDL1–2) [20].

Nedd4-1, highly expressed in placenta, liver, thyroid, skin, and kidney, modulates embryonic development and growth predominantly through K48- and K63-linked polyubiquitination, with minor contributions from mono-ubiquitination and K6/K27 linkages [20], [61]. It recognizes a proline-rich sequence in α-syn and is upregulated in Lewy body-containing neurons [61]. Paradoxically, while *Nedd4-1* deficiency reduces α-syn degradation, it primarily mono-ubiquitinates or K63-polyubiquitinates α-syn, thereby redirecting it to endolysosomal rather than proteasomal degradation [20]. Nedd4-2 ubiquitinates the calcium channel Orai calcium release-activated calcium modulator 1 (ORAI1), targeting it for degradation, a process inhibited by serum/glucocorticoid-regulated kinase 1 (SGK1)-mediated phosphorylation, which promotes protein 14-3-3 binding and blocks Nedd4-2-membrane protein interactions [62].

Furthermore, ITCH/AIP4 terminates inflammatory signaling and antiviral responses [63], whereas the WWP and Smurf subfamily members operate through distinct mechanisms: WWP1 drives the enhancer of split and hairy-related protein 1 (SHARP1) degradation in metabolic dysfunction-associated steatotic liver disease (MASLD), linking ubiquitination to lipid metabolism and inflammation [64], [65], and Smurf1 activates transcription factor EB (TFEB)-mediated lysosome biogenesis by ubiquitinating protein phosphatase 3 catalytic subunit β (PPP3CB), requiring coordination with lectin galectin-3 (LGALS3) and protein phosphatase 3 regulatory subunit 1 (PPP3R1) during lysosomal damage responses [66]. Smurf2 colocalizes with phospho-tau inclusions in progressive supranuclear palsy (PSP) [67], and its downregulation leads to hyperactivation of transforming growth factor-β observed in AD, PD, and ALS [68]. NEDL1 contributes to ALS pathology through defective protein degradation, TDP-43 mislocalization, and neuronal death [64]. WWP2 and NEDL2 were excluded from this review due to a lack of direct ND evidence, WWP2 functionally overlaps with WWP1 and has been primarily studied in cancer, whereas NEDL2 research remains in early stages, with only developmental roles reported [68].

#### 3.4.2 HERC

HERCs have N-terminal RLDs that function as Ran GTPase guanine nucleotide exchange factors and bind to chromatin through histones H2A/H2B [66]. When subclassified by RLD number, large HERCs, such as HERC2, regulate E6AP activity; mutations in HERC2 that cause its degradation can lead to an Angelman syndrome (AS)-like phenotype [69].

#### 3.4.3 E6-associated protein

The first HECT E3 ligase discovered, E6AP [encoded by *UBE3A* (ubiquitin protein ligase E3A)], contains an N-terminal amino-terminal Zn-finger (AZUL) domain for substrate recognition and autoinhibition. It mainly assembles K48-linked polyubiquitin chains and is linked to autism (via *HECT* domain mutations) and neurodegeneration [knockdown speeds up polyQ (polyglutamine) aggregate toxicity] [70]. In AS, restoring paternal *UBE3A* expression with topoisomerase inhibitors (e.g., topotecan) or antisense oligonucleotides (ASOs) improves cognitive deficits in mouse models [71], [72]. E6AP also reduces mutant huntingtin (mHtt) aggregation in HD [70].

E6AP-related therapeutic strategies aim to reverse *UBE3A* silencing. For example, introducing topoisomerase inhibitors, such as topotecan, restored *UBE3A* expression from the paternal allele [73]. Gene therapies, such as ASOs, were shown to unsilence neuronal paternal *UBE3A* and ameliorate certain cognitive deficits (e.g., freezing behavior) in AS model mice, without affecting other behavioral phenotypes [71]. E6AP has also been shown to suppress mHtt aggregation in HD and decrease cellular toxicity [70].

## 4 E3 Ub ligases in neurodegenerative diseases

A central pathological hallmark unifying many NDs is the aberrant accumulation of misfolded proteins into toxic aggregates. These include intracellular Lewy bodies composed primarily of α-syn in PD [74], [75] and in the oligodendrocytes of MSA, where they form glial cytoplasmic inclusions (GCIs) [76]; extracellular Aβ plaques and intracellular neurofibrillary tangles of hyperphosphorylated tau in AD [77], [78] and cytoplasmic inclusions of TDP-43 or superoxide dismutase 1 (SOD1) in ALS, as well as cytoplasmic and nuclear aggregates of mHtt in HD [79], [80], [81]. These pathological proteins often exhibit prion-like properties, propagating disease pathology by seeding the misfolding of native, soluble proteins [75], [77]. A critical downstream effect of this proteostasis failure is severe mitochondrial dysfunction, in which proteins such as α-syn can impair complex I activity, thereby increasing oxidative stress and neuronal vulnerability [82], [83]. While most cases are idiopathic, familial forms provide crucial insights, often stemming from mutations in genes encoding proteins vital to protein quality control, including several E3 Ub ligases like *Parkin (PRKN)* in PD [84], or from repeat expansions such as the *C9orf72* (chromosome 9 open reading frame 72) hexanucleotide repeat in a significant portion of ALS and frontotemporal dementia (FTD) cases [81], [85]. The consequent dysfunction of the UPS and autophagy-lysosomal pathways, which are essential for clearing damaged proteins and organelles, is a common topic, as evidenced by the frequent presence of Ub within pathological inclusions such as Lewy bodies, neurofibrillary tangles, and TDP-43-positive aggregates [79], [86], [87]. Thus, the core pathophysiology of NDs revolves around a self-reinforcing cycle of proteostasis failure, bioenergetic deficit, and often neuroinflammation, ultimately triggering selective neuronal death.

### 4.1 Mitophagy-regulating E3 ligases

#### 4.1.1 Parkin (*PRKN*): the PINK1-Parkin pathway

Parkin functions as an RBR E3 ligase that is pivotal for mitochondrial quality control, and pathogenic mutations in the *PRKN* gene are the most common cause of autosomal recessive familial PD [84]. Parkin activation follows a well-defined phosphorylation cascade [88]. Firstly, mitochondrial depolarization stabilizes PINK1 on the outer mitochondrial membrane, where it phosphorylates free ubiquitin at Ser65 (p-Ub). Secondly, p-Ub binds Parkin’s RING0 domain, allosterically disrupting the REP-RING1 interface to enable PINK1-mediated phosphorylation of Parkin’s Ubl domain at Ser65 (p-Parkin). This p-Ub to p-Parkin relay fully activates the ligase, thereby recruiting E2. Activated Parkin then ubiquitinates outer membrane, matrix, and inner membrane proteins, generating phospho-ubiquitin chains that recruit autophagy receptors (optineurin, Rab proteins) and engage LC3 for lysosomal degradation [88].

The pathogenic consequences of Parkin dysfunction are multifaceted. In PD, *PRKN* mutations impair mitophagy, leading to dopaminergic neuron loss in the substantia nigra pars compacta (SNpc) and locus coeruleus [89]. Additionally, S-nitrosylation of Parkin promotes its aggregation into Lewy bodies, diminishing the pool of soluble Parkin available for neuroprotective functions [90]. In induced pluripotent stem cell (iPSC)-derived neurons with *Parkin* deficiency, α-syn aggregation and seeding competence are markedly enhanced [91]. Parkin also directly modulates α-syn pathology through multiple mechanisms: it inhibits Polo-like kinase 2-mediated Ser129 phosphorylation via activation of PP2A [92], [93]; directly ubiquitinates the 22-kD glycosylated form of α-syn (αSp22) [94]; and targets the α-syn interactor synphilin-1, licensing formation of K63-Ub-positive inclusions that sequester toxic oligomers for autophagic clearance, a function crippled by familial *Parkin* mutations [95]. Additionally, Parkin activates PP2A to dephosphorylate Ser129 of α-syn, reducing oligomer accumulation and Lewy body formation [92], [93]. Therapeutic activation of the PINK1/Parkin pathway shows promise in PD models. *Salvia miltiorrhiza* Bunge activates PINK1/Parkin-dependent mitophagy and reduces oxidative stress; Similarly, Epicoccin A, a metabolite from *Exserohilum* sp., exerts neuroprotective effects through the same mechanism [96], [97].

Beyond PD, Parkin’s neuroprotective roles extend to AD, where it reduces tau hyperphosphorylation by downregulating glycogen synthase kinase 3β (GSK-3β) and upregulating PP2A [98]. Multiple therapeutic studies demonstrate that natural compounds, including *Curcuma wenyujin* extract, β-asarone, and tetrahydroxy stilbene glycoside, as well as dual cholecystokinin (CCK)/GLP-1 receptor agonists, ameliorate AD pathology by enhancing PINK1/Parkin-mediated mitophagy [99], [100], [101], [102]. In MSA, Parkin has been identified in GCIs with disease-specific splice isoforms, broadening its pathological relevance beyond PD. The *TV2* variant is elevated in MSA brains compared with PD and controls, whereas *TV12* is more abundant in the MSA striatum and cerebellar cortex. Notably, isoforms lacking Ub-like domains (*TV6*, *TV7*, *TV12*) exhibit diminished proteasomal degradation efficacy, potentially compromising Parkin’s neuroprotective capacity in MSA [86].

#### 4.1.2 F-box only protein 7: PINK1-Parkin recruitment factors

F-box only protein 7 (FBXO7), an F-box subunit of SCF E3 ligases, is essential for recruiting Parkin during mitophagy, and loss-of-function mutations lead to early-onset autosomal recessive PD [103]. The protein interacts with PINK1 and collaborates with Parkin to initiate downstream mitophagy processes. *FBXO7* knockout mice show accumulation of fragmented mitochondria in dopaminergic and cortical neurons, along with Lewy body-like aggregates containing p62 and α-syn, resulting in locomotor impairment and a reduction of tyrosine hydroxylase-positive neurons in the SNpc [103]. Under oxidative stress, mutant *FBXO7* forms aggregates in mitochondria and nuclei, disrupting carbonyl cyanide-4-c(trifluoromethoxy)phenylhydrazone (FCCP)-induced mitophagy and increasing reactive oxygen species (ROS) production [104]. *In vitro* studies reveal that *FBXO7* mutations in SK-N-SH neuroblastoma cells cause cytoplasmic aggregation of both FBXO7 and Parkin. When exposed to oxidative stress, such as H_2_O_2_ treatment, mutant *FBXO7* aggregation worsens in both mitochondrial and nuclear compartments, leading to impaired mitochondrial function, increased ROS levels, and decreased clearance of damaged mitochondria [104]. These findings identify FBXO7 as an important upstream regulator whose dysfunction promotes mitochondrial stress and protein aggregation in PD pathogenesis.

While current evidence for FBXO7 dysfunction is mainly limited to PD pathogenesis, its key role as a gatekeeper for Parkin recruitment makes it a potential modifier in other NDs marked by mitochondrial quality-control failure. However, since mitophagy dysfunction is a common mechanism across NDs, FBXO7-mediated regulation could impact disease progression where Parkin-dependent clearance is impaired, though this remains speculative until proven by experimental evidence.

### 4.2 Tripartite motif-containing protein 31: voltage-dependent anion channel 1 degradation

Tripartite motif-containing protein 31 (TRIM31), a TRIM family E3 ligase (Table 2 [105], [106], [107], [108], [109], [110]), protects dopaminergic neurons by specifically targeting voltage-dependent anion channel 1 (VDAC1) for K48-linked polyubiquitination and proteasomal degradation [111]. *TRIM31*-deficient mice can develop age-related motor deficits and nigrostriatal degeneration accompanied by mitochondrial abnormalities [111]. Lower TRIM31 expression in PD models correlates with elevated VDAC1 levels, promoting the opening of mitochondrial permeability transition pore and neuronal death. Conversely, TRIM31 overexpression mitigates neurotoxicity by enhancing VDAC1 degradation [111]. Besides its role in neurodegeneration, TRIM31 also regulates viral infections and hypertensive nephropathy by ubiquitinating specific target proteins [112], [113], highlighting its diverse functions in cellular stress responses. Identifying TRIM31 as a key regulator of mitochondrial function makes it a promising therapeutic target for protecting dopaminergic neurons in PD by maintaining mitochondrial integrity.

**Table 2:** TRIM family in neurodegeneration.

**TRIM**	**Mechanisms**	**Role in NDs**	**Key findings**	**References**
TRIM11	Molecular chaperone/disaggregase	Prevents and reverses protein aggregation in PD and SCA1	Protects substrates from thermal misfolding and aggregation	[110]
TRIM19/PML	SUMOylation and ubiquitination	Degrades nuclear protein aggregates in HD, ALS, and FTD	SUMOylates misfolded proteins	[105]
TRIM21	Ubiquitination and autophagy	Degrades tau aggregates in AD	Detects antibody-bound tau proteins	[110]
TRIM8, TRIM22, TRIM36	Ubiquitination	Promotes degradation of misfolded proteins	Elevated expression during oncogenic transformation	[105]
TRIM5α	Autophagy receptor	Initiates autophagy and clears protein aggregates	Acts as a scaffold for ULK1 and Beclin1	[105]
TRIM16	Autophagy and ubiquitination	Promotes autophagic degradation of protein aggregates	Interacts with p62, ULK1, ATG16L1, and LC3	[106]
TRIM17	Ubiquitination	Regulates α-syn expression in PD	Stabilizes ZSCAN21 to promote SNCA transcription	[107]
TRIM28	Ubiquitination	Promotes nuclear accumulation of α-syn and tau in PD and AD	Drives pathogenic protein accumulation	[108], [109]
TRIM41	Ubiquitination	Regulates α-syn expression in PD	Ubiquitinates ZSCAN21 for degradation	[107]

TRIM. Tripartite motif; PML. Promyelocytic leukemia (protein); ULK1. Unc-51-like autophagy activating kinase 1; ATG16L1. Autophagy-related 16-like 1; ZSCAN21. Zinc finger and SCAN domain containing 21; SCA1. Spinocerebellar ataxia type 1; LC3. Microtubule-associated protein 1A/1B-light chain 3; NDs. Neurodegenerative diseases; PD. Parkinson’s disease; HD. Huntington’s disease; ALS. Amyotrophic lateral sclerosis; FTD. Frontotemporal dementia; AD. Alzheimer’s disease; SNCA. Synuclein alpha

### 4.3 Protein quality control: CHIP/STIP1 homology and U-box containing protein 1 (STUB1)

CHIP [or STIP1 homology and U-box containing protein 1 (STUB1)] integrates chaperone and UPS functions through its TPR domain (which binds HSP70/90) and its U-box (E3 activity), positioning it as an important hub for proteostasis across multiple NDs. CHIP’s core mechanism involves recognizing misfolded proteins, mediating K48-linked ubiquitination for proteasomal degradation, and indirectly regulating HSP70 stability to clear toxic aggregates. In PD, CHIP compensates for Parkin loss by enhancing ubiquitination of misfolded proteins and damaged mitochondria [114]. It preferentially targets toxic α-syn oligomers for both proteasomal and lysosomal degradation pathways [115] and directly ubiquitinates leucine-rich repeat kinase 2 (LRRK2), rescuing mitochondrial defects [116]. Notably, CHIP also targets PINK1, promoting its ubiquitination and degradation in *PINK1* mutants [117], [118].

In AD, CHIP overexpression reduces Aβ production by degrading β-site amyloid precursor protein (APP)-cleaving enzyme 1 (BACE1) via UPS [119]. It also reduces tau-associated mitochondrial damage and transport deficits in neuronal models [119]. The cytosolic region of APP interacts directly with the Cullin-RING ligase 4 (CRL4)-type E3 Ub ligase [cereblon (CRBN)], facilitating the ubiquitination of presynaptic proteins [120]. Furthermore, upregulation of methyltransferase 3, N^6^-adenosine-methyltransferase complex catalytic subunit (*METTL3*), enhances CHIP (or STUB1)-mediated autophagic clearance of phosphorylated tau through m^6^A-dependent mechanisms [121]. CHIP binds phosphorylated tau with 10-fold higher affinity than unmodified tau, suppressing aggregation and promoting rapid ubiquitination at sub-stoichiometric concentrations [122]. *STUB1* variants are also associated with cerebellar ataxia and cognitive decline [123], [124].

In HD, CHIP reduces mHtt aggregation by promoting HSP70-dependent refolding rather than UPS degradation, and co-overexpression of HSP70 further enhances neuroprotection [125], [126], [127]. In ALS, CHIP does not directly ubiquitinate mutant *SOD1* but instead polyubiquitinates HSP70, leading to the degradation of the HSP70-SOD1 complex by the proteasome [87]. Therapeutically, HSP70 activators such as YM-1 and MKT-077 stabilize the ADP-bound form of HSP70, thereby enhancing its interaction with CHIP and increasing ubiquitination and proteasomal degradation of misfolded proteins in ALS and other ND models [128].

### 4.4 Endoplasmic reticulum (ER)-associated degradation E3 ligases

#### 4.4.1 GP78: ER-mitochondria cross-talk

GP78, an ER-anchored E3 ligase also known as autocrine motility factor receptor, contains 5 transmembrane domains and a cytoplasmic C-terminus with RING finger, coupling of Ub to ER degradation motif (CUE), GP78 2-binding region (G2BR), and Vimentin (VIM) domains [125]. In PD, cyclin-dependent kinase 5 (CDK5) phosphorylates GP78 at Ser516, leading to its ubiquitin-proteasome degradation. GP78 levels are low in PD models, and blocking its phosphorylation helps protect neurons from 1-methyl-4-phenylpyridinium (MPP^+^)-induced death. Additionally, GP78 ubiquitinates mitofusins (Mfn)1 and Mfn2 for proteasomal degradation, inducing mitochondrial fragmentation and Parkin-independent mitophagy by recruiting LC3 to mitochondria-associated ER membranes [126]. While GP78’s pathogenic role is primarily documented in PD, its fundamental role in ER-associated degradation (ERAD) and mitochondrial quality control suggests potential relevance to other NDs in which ER stress and mitochondrial dysfunction converge, such as AD and ALS/FTD, though specific mechanisms in these contexts remain to be experimentally validated.

#### 4.4.2 HMG-CoA reductase degradation protein 1: ERAD-mediated clearance of misfolded proteins

**Fig. 3:**
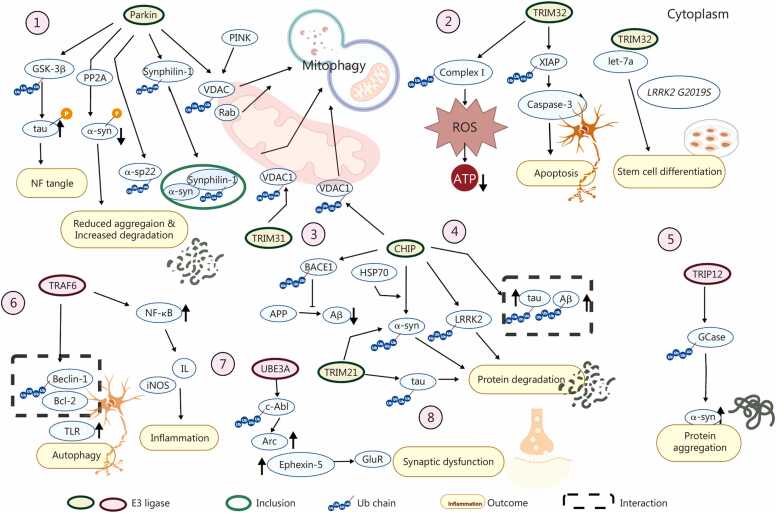
Pathways of E3 ubiquitin ligases in neurodegenerative diseases. E3 ubiquitin ligases play a crucial role in the onset and progression of neurodegenerative disorders. Eight E3 ligases (Parkin, CHIP, TRIM32, TRIM21, TRIM31, TRIP12, TRAF6, and UBE3A) directly conjugate ubiquitin to substrates, tagging the substrates for degradation and coordinating essential biological or pathological processes: ① Parkin inhibits tau hyperphosphorylation in amyloid-stressed M17 neuroblastoma cells when GSK-3β activity is low and activates PP2A to dephosphorylate Ser129 α-syn, thereby reducing oligomer buildup and Lewy body formation. It ubiquitinates the 22-kD glycosylated α-syn species (αSp22) and links synphilin-1 with K63-linked chains to form inclusions that trap toxic α-syn oligomers and direct them to autophagy. Alongside PINK1, Parkin ubiquitinates VDAC, recruiting Rab proteins and LC3 to promote mitophagy. ② TRIM32 decreases XIAP, thereby activating Caspase-3 and promoting apoptosis. It opposes pathogenic LRRK2-mediated repression of let-7a, facilitating neuronal differentiation in human neuroepithelial stem cells. Under stress, TRIM32 relocates to the outer mitochondrial membrane, disrupts complex I, increases ROS, and reduces ATP. ③ TRIM31 conjugates K48-linked polyubiquitin to VDAC1, directing its proteasomal degradation. ④ CHIP co-localizes with α-syn and HSP70 in Lewy bodies; its overexpression reduces α-syn aggregation and accelerates its turnover. CHIP also decreases BACE1 levels by increasing UPS-mediated degradation, thus reducing APP processing and Aβ production and aiding in the clearance of Aβ42-associated tau pathology. Additionally, CHIP binds to, ubiquitinates, and facilitates the degradation of LRRK2. ⑤ TRIP12 ubiquitinates GCase at Lys293, leading to its proteasomal degradation, impairing mitochondrial function, and promoting α-syn accumulation. ⑥ TRAF6 recruits NIK to the IKK signalosome, preventing IκB degradation and enabling IκB phosphorylation. Separately, TRAF6 forms K63-linked ubiquitin chains on Beclin-1, decreasing its interaction with Bcl-2 and amplifying TLR-induced autophagy. ⑦ UBE3A ubiquitinates the synaptotoxic factors Arc and Ephexin-5, counteracting synaptic impairment. ⑧ TRIM21 mediates antibody-dependent clearance of tau aggregates, reducing tau pathology in mice, and targets α-syn for degradation, alleviating its aggregation. CHIP. C-terminus of HSC70-interacting protein; TRIM. Tripartite motif-containing protein 31; TRIP12. Tumor necrosis factor receptor-associated factor-interacting protein 12; TRAF6. Tumor necrosis factor receptor-associated factor 6; UBE3A. Ubiquitin ligase E3A; LC3. Microtubule-associated protein 1 A/1B-light chain 3; PP2A. Protein phosphatase 2A; GSK-3β. Glycogen synthase kinase 3β; α-syn. α-synuclein; VDAC. Voltage-dependent anion channel; XIAP. X-linked inhibitor of apoptosis protein; LRRK2. Leucine-rich repeat kinase 2; ROS. Reactive oxygen species; BACE1. Beta-secretase 1; APP. Amyloid precursor protein; Aβ. Amyloid-beta; UPS. Ubiquitin-proteasome system; GCase. Glucocerebrosidase; NF-κB. Nuclear factor κB; NIK. NF-κB-inducing kinase; IKK. IκB kinase; IκB. Inhibitor of kappa B; TLR. Toll-like receptor; Arc. Activity-regulated cytoskeleton-associated protein.

HMG-CoA reductase degradation protein 1 (HRD1) is an ERAD E3 ligase that forms retro-translocation pores and ubiquitinates misfolded proteins for p97/VCP-mediated extraction. In HD, HRD1 auto-ubiquitinates, creating high-affinity binding sites for Htt and ubiquitinating both wild-type and mutant forms, thereby promoting proteasomal degradation. HRD1 overexpression reduces mHtt aggregation and toxicity, with greater effectiveness for longer polyQ repeats [127]. However, the current evidence does not directly confirm HRD1’s involvement outside HD, underscoring an important area for future studies, whether increasing HRD1 levels could reduce proteotoxicity in these related disorders. The roles of E3 ligases in NDs are summarized in Fig. 3.

### 4.5 Disease-specific E3 ligases

#### 4.5.1 Parkinson’s disease-specific regulators

Thyroid hormone receptor-interacting protein 12 (TRIP12) functions as a key regulator of lysosomal activity in PD by managing glucosylceramidase (GCase), this HECT tags GCase at Lys293 with Ub, leading to its degradation [128]. In PD brains, elevated TRIP12 levels correlate with decreased GCase activity and increased loss of dopaminergic neurons, creating a harmful feedback loop that worsens α-syn accumulation. Notably, reducing TRIP12 levels in both *in vitro* and *in vivo* models restores GCase activity, diminishes α-syn-related damage, and shields dopaminergic neurons from degeneration [128]. In addition to its role in maintaining lysosomal health, TRIP12 also regulates the oxidative stress response by cooperating with CUL3^KEAP1^ to promote NRF2 degradation, thereby limiting cellular antioxidant defenses [129]. This dual role, which disrupts lysosomal function and reduces antioxidant capacity, makes TRIP12 a promising target for therapies aimed at restoring cellular balance in PD [129].

TRIM32 is another PD-related E3 ligase with multiple roles in disease [130]. Under cellular stress, TRIM32 translocates to the outer mitochondrial membrane via its RING domain, impairing complex I function, increasing mitochondrial ROS production, and lowering ATP levels [131]. TRIM32 also promotes apoptosis by tagging and degrading the X-linked inhibitor of apoptosis protein (XIAP), an effect that can be blocked by coexpressing XIAP or mitochondrial-targeted antioxidants [131]. At the molecular level, TRIM32 forms a complex with LRRK2 and Argonaute 2 (Ago2), counteracting harmful *LRRK2* mutations (*G2019S* and *R1441H*) that inhibit let-7a miRNA activity [132]. This indicates TRIM32 might oppose LRRK2-driven pathology. Additionally, TRIM32 acts as a transcriptional regulator by binding to the *SNCA* promoter and influencing α-syn expression during neuronal differentiation, with *SNCA* decreased in the olfactory bulb of *TRIM32*-deficient mice [133]. These diverse mechanisms implicate TRIM32 in mitochondrial dysfunction and neurodevelopmental processes underlying PD pathogenesis.

Other members of the TRIM family, such as TRIM21, help break down tau aggregates via antibody-dependent mechanisms and aid in clearing α-syn, thereby reducing pathology in mouse models [134]. Conversely, a loss-of-function mutation in *TRIM25* (p.C168*) causes early-onset autosomal dominant dementia with amyloid deposits and Parkinsonism [135], highlighting the complexity of the TRIM family in neurodegeneration.

#### 4.5.2 Alzheimer’s disease-specific regulators

FBXO2, an F-box component of SCF E3 ligases, selectively targets high-mannose glycoproteins for degradation and plays a key role in AD pathogenesis by regulating APP processing. FBXO2 ubiquitinates and degrades the APP-cleaving enzyme BACE1, thereby decreasing Aβ production [136]. In *FBXO2*-knockout mice and cultured hippocampal neurons, APP levels and cleavage products are significantly increased, while *FBXO2* upregulation reduces BACE1 and Aβ [137]. Importantly, FBXO2 expression declines with age in the brains of AD patients, indicating that *FBXO2* deficiency worsens amyloid pathology [137].

Tumor necrosis factor receptor-associated factor 6 (TRAF6) acts as both an adaptor protein and an E3 ligase, linking immune signaling to autophagy in AD. Toll-like receptor (TLR) 4 activation initiates TRAF6-mediated K63 ubiquitination of Beclin-1 at Lys117, which boosts phosphatidylinositol 3-kinase catalytic subunit type 3 (PI3KC3) enzymatic activity and encourages autophagosome formation [138]. In astrocytes, TRAF6 drives detrimental neuroinflammatory responses by recruiting NF-κB-inducing kinase (NIK) to the IκB kinase signalosome, activating NF-κB upon Aβ-42 stimulation. This cascade induces inducible nitric oxide synthase (iNOS) and exacerbates inflammatory damage [139].

Membrane-associated ring-CH-type finger 5 (MARCHF5), a mitochondrial E3 ligase involved in mitochondrial fusion and fission dynamics, protects against AD pathology. Knockout of *MARCHF5 in vivo* causes irregular mitochondrial morphology, speeds up Aβ fibril seeding [140], and increases the production of toxic off-pathway Aβ oligomers. Patients show reduced *MARCHF5* expression, linking mitochondrial quality control to amyloid toxicity in AD [140].

UBE3A/E6AP, although primarily recognized for its role in AS, also contributes to synaptic pathology in AD. Aβ oligomers recruit the non-receptor tyrosine kinase cellular Abelson tyrosine kinase (c-Abl), which inhibits UBE3A activity and reduces its half-life, thereby promoting the accumulation of synaptotoxic substrates such as activity-regulated cytoskeleton-associated protein (Arc) and Ephexin-5 [126]. This process decreases the surface expression of the α-amino-3-hydroxy-5-methyl-4-isoxazolepropionic acid (AMPA) receptor subunit GluR1 and increases RhoA activity, resulting in dendritic spine pruning and impaired synaptic function [126].

#### 4.5.3 Huntington’s disease-specific regulators

Protein inhibitor of activated STAT 1 (PIAS1), a small ubiquitin-like modifier (SUMO) E3 ligase for the DNA repair enzyme PNKP, modulates transcription-coupled repair and protein homeostasis in HD. In HD models, *PIAS1* knockdown or genetic reduction via the protective variant *PIAS1(S510G)* normalizes transcriptional dysregulation, restores PNKP activity, enhances genomic integrity, reduces mHtt aggregation and SUMOylation, and ultimately improves motor function while attenuating neuroinflammation [141], [142], [143].

Additionally, WWP1 contributes to HD pathogenesis through a gain-of-toxicity mechanism. In HD mouse models and mHtt-expressing cells, elevated WWP1 co-localizes with aggregates and promotes neuronal toxicity [79]. Mechanistically, WWP1 ubiquitinates mHtt at Lys63, generating non-degradative Ub chains that inhibit proteasomal degradation and enhance aggregation [79]. This atypical ubiquitination pattern distinguishes WWP1’s pathogenic role in HD from conventional degradative ubiquitination.

Htt is highly expressed in primate striatal neurons due to its slow degradation in this brain region. TRIM37, a primate-specific E3 ligase that selectively targets Htt and promotes its ubiquitination and degradation, is specifically reduced in the primate striatum compared to other brain regions. This regional expression disparity impairs mutant Htt ubiquitination and clearance, thereby exacerbating the selective vulnerability of striatal neurons in HD [144]. This species- and region-specific expression pattern may explain the unique susceptibility of striatal neurons in HD.

F-box and WD repeat domain containing 7 (FBXW7), together with casein kinase 2α (CK2α), forms a regulatory axis that controls the stability of heat shock transcription factor 1 (HSF1). Elevated CK2α and FBXW7 phosphorylate HSF1, promoting its proteasomal degradation. Reducing CK2α expression in HD mouse models increases HSF1 levels, reduces mHtt aggregates, and preserves body mass, suggesting that modulating this axis could restore proteostasis in HD [145].

#### 4.5.4 Multiple system atrophy-specific regulators

Siah E3 ubiquitin protein ligase 1 [seven in absentia homolog 1 (SIAH-1)], a RING-type E3 ligase, plays divergent pathogenic roles in MSA and PD. In MSA, SIAH-1 accumulates in GCIs and exerts a toxic gain-of-function by aberrantly mono- and di-ubiquitinating α-syn, thereby promoting its aggregation and insolubility rather than proteasomal clearance [146], [147]. Concurrently, SIAH-1-mediated degradation of the presynaptic protein synaptophysin exacerbates neuronal dysfunction; together, these mechanisms drive glial α-syn pathology and apoptosis in MSA [146], [147].

#### 4.5.5 Amyotrophic lateral sclerosis/frontotemporal dementia-associated E3 ligases

Ring finger protein 220 (RNF220) polyubiquitinates TDP-43 via K48 linkages, targeting it for proteasomal degradation. When RNF220 levels are reduced (haploin-sufficiency), this clearance mechanism fails, resulting in TDP-43 aggregation specifically in spinal motor neurons and causing ALS-like motor neurotoxicity [148]. In contrast to this loss-of-function mechanism, Cyclin F (CCNF), an F-box protein in the SCF complex, regulates TDP-43 and E2F1 degradation [149], [150]. ALS/FTD-linked mutant *CCNF* (p.S621G) aberrantly ubiquitinates p62 at Lys281, reducing its insolubility and disrupting p62 foci formation. This variant also impairs UPS function, leading to increased ubiquitinated proteins in iPSC-derived motor neurons [151], [152]. Additionally, CCNF regulates p62 aggregation and increases Lys48-linked ubiquitination of proteins, with the pathogenic mutation disrupting these protective functions [153]. Similarly, gain-of-function mechanisms contribute to motor neuron pathology. NEDL1, encoded by the *HECW1* gene, causes motor neuron degeneration when overexpressed. Human *NEDL1* transgenic mice exhibit muscle atrophy, motor neuron degeneration, and increased numbers of activated microglia in the lumbar spinal cord, with TDP-43 mislocalization, suggesting relevance to familial ALS pathology [64], [154]. Conversely, some E3 ligases employ alternative ubiquitination strategies for neuroprotection. Smurf1 promotes K63-linked ubiquitination of misfolded SOD1, thereby encouraging aggresome formation and autophagic clearance, thereby reducing cellular toxicity [80]. This alternative ubiquitination method is a protective mechanism separate from degradative K48 linkages. See Table 3 [50], [52], [58], [64], [70], [71], [73], [80], [84], [86], [89], [91], [92], [103], [104], [111], [112], [115], [117], [119], [128], [131], [132], [133], [134], [137], [138], [139], [140], [146], [147], [148], [149], [150], [154], [155] for the full information.

**Table 3:** E3 ubiquitin ligase in neurodegenerative diseases.

**Disease**	**E3 ligase**	**Family**	**Mechanism**	**Substrate**	**Consequence**	**Therapeutics**	**References**
AD	CHIP/STUB1	U-box	Tau & p-tau ubiquitination; BACE1 degradation	p-tau, BACE1	Variants cause ataxia; Reduces Aβ & tau pathology	miR-9 inhibition, HSP70 activators	[119]
	TRIM21	TRIM	Antibody-dependent tau aggregate degradation	Tau aggregates	Attenuates tau pathology in mice	Immunotherapy combination	[50], [134]
	FBXO2	SCF (F-box)	BACE1 degradation	BACE1	Deficiency → ↑Aβ production	Gene therapy, stabilizers	[137]
	UBE3A/E6AP	HECT	Aβ oligomers inhibit UBE3A → synaptic toxicity	Arc, Ephexin-5	Spine pruning, synaptic impairment	UBE3A restoration (topotecan)	[70], [71], [73]
	MARCHF5	MARCH	Mitochondrial fusion/fission regulation	Mitochondrial proteins	Reduction → Aβ oligomer accumulation	MARCHF5 activators	[140]
	TRAF6	TRIM	Neuroinflammation; Beclin-1 K63-ubiquitination	Beclin-1, NF-κB-inducing kinase	NFκB activation, microglial inflammation	TRAF6 inhibitors	[138], [139]
	TRIP12	HECT	Oxidative stress dysregulation (NRF2 degradation)	NRF2	Impaired antioxidant response	TRIP12 inhibitors	[128]
PD	Parkin/PRKN	RBR	PINK1-Parkin mitophagy; α-syn regulation (αSp22, synphilin-1)	OMM proteins, α-syn	Mutations → mitophagy failure, α-syn aggregation	PINK1 activators, PROTACs	[58], [84], [89], [91], [92]
	FBXO7	SCF (F-box)	Parkin recruitment during mitophagy	Parkin	Loss-of-function → early-onset PD, Lewy bodies	Mitophagy enhancers	[103], [104]
	CHIP/STUB1	U-box	Compensates Parkin α-syn oligomer clearance	α-syn, LRRK2	Reduces toxic oligomers	HSP70 activators	[52], [117]
	TRIM31	TRIM	VDAC1 K48-ubiquitination & degradation	VDAC1	Deficiency → mitochondrial dysfunction, neuron loss	*TRIM31* gene therapy	[111], [112]
	TRIM21	TRIM	Antibody-dependent α-syn clearance	α-syn	Reduces Lewy pathology	Immunotherapy combination	[134]
	TRIM32	TRIM	Mitochondrial translocation → Complex I dysfunction	XIAP, LRRK2	ROS↑, ATP↓, apoptosis activation	TRIM32 inhibitors	[131], [132], [133]
	TRIP12	HECT	GCase ubiquitination (Lys293) → lysosomal dysfunction	GCase	↓GCase activity → α-syn accumulation	*TRIP12* knockdown	[128]
	SIAH-1	RING	α-syn mono/di-ubiquitination → insolubility	α-syn	Promotes glial aggregation (MSA-specific)	SIAH-1 inhibitors	[146], [147]
ALS	HECW1/NEDL1	HECT	TDP-43 mislocalization; motor neuron degeneration	TDP-43, SOD1	Pathogenic variants → TDP-43 pathology, microglial activation	*HECW1* knockdown	[64], [154]
	RNF220	RING	TDP-43 K48-polyubiquitination & proteasomal degradation	TDP-43	Haploinsufficiency → TDP-43 aggregation, motor neuron loss	*RNF220* gene therapy	[148]
	CCNF/Cyclin F	SCF (F-box)	Aberrant p62 ubiquitination (p.S621G) UPS impairment	p62, E2F1	Mutant disrupts protective functions → protein aggregation	CCNF correctors	[149], [150]
	CHIP/STUB1	U-box	HSP70-SOD1 complex ubiquitination & degradation	HSP70-SOD1 complex	Reduces misfolded SOD1 toxicity	HSP70 activators	[52], [155]
	Smurf1	HECT	K63-ubiquitination → aggresome formation & autophagic clearance	Misfolded SOD1	Reduces cellular toxicity via alternative pathway	Smurf1 activators	[80]
MSA	Parkin isoforms (TV2, TV6, TV7, TV12)	RBR	Disease-specific splice variants lacking Ubl domains	Autoubiquitination	Diminished proteasomal efficacy, compromised neuroprotection	Isoform-specific targeting	[86]
	SIAH-1	RING	α-syn mono/di-ubiquitination in oligodendrocytes	α-syn	Promotes GCI formation	SIAH-1 inhibitors	[146], [147]
	CHIP/STUB1	U-box	General protein quality control (overlaps with PD/ALS)	Misfolded proteins	Reduces α-syn oligomerization	HSP70 activators	[115]

AD. Alzheimer’s disease; PD. Parkinson’s disease; ALS. Amyotrophic lateral sclerosis; MSA. Multiple system atrophy; CHIP/STUB1. C-terminus of HSC70-interacting protein/STIP1 homology and U-box containing protein 1; TRIM. Tripartite motif; TRIM19/PML. Tripartite motif containing 19/promyelocytic leukemia; FBXO2. F-box protein 2; FBXO7. F-box protein 7; SCF. Skp1-Cullin1-F-box; UBE3A/E6AP. Ubiquitin protein ligase E3A/E6-associated protein; MARCHF5. Membrane-associated RING-CH-type finger 5; MARCH. Membrane-associated RING-CH; TRAF6. TNF receptor-associated factor 6; TRIP12. Thyroid hormone receptor interactor 12; RBR. RING-between-RING; RING. Really interesting new gene; HECT. Homologous to the E6AP carboxyl terminus; SIAH-1. Seven in Absentia homolog 1; HECW1/NEDL1. HECT, C2 and WW domain containing E3 ubiquitin protein ligase 1/neural precursor cell expressed, developmentally down-regulated 4-like 1; RNF220. Ring finger protein 220; CCNF. Cyclin F; Smurf1. SMAD-specific E3 ubiquitin protein ligase 1; BACE1. Beta amyloid precursor protein cleaving enzyme 1; SOD1. Superoxide dismutase 1; TDP-43. TAR DNA-binding protein 43; LRRK2. Leucine-rich repeat kinase 2; GCase. Glucocerebrosidase; VDAC1. Voltage-dependent anion channel 1; XIAP. X-linked inhibitor of apoptosis protein; NRF2. Nuclear factor erythroid 2-related factor 2; Arc. Activity-regulated cytoskeleton-associated protein; NF-κB. Nuclear factor κB; OMM. Outer mitochondrial membrane; PINK1. PTEN-induced putative kinase 1; HSP70. Heat shock protein 70; STIP1. Stress-induced phosphoprotein 1; GCI. Glial cytoplasmic inclusion; UPS. Ubiquitin-proteasome system; ROS. Reactive oxygen species; ATP. Adenosine triphosphate; PROTAC. Proteolysis targeting chimera; Ubl. Ubiquitin-like domain

#### 4.5.6 Rare variants and emerging targets

Gu et al. [156] analyzed 695 early-onset PD patients to investigate the enrichment of rare variants in E3 Ub ligase genes and found significant enrichment of rare and damaging variants. The strongest associations were observed in genes such as *HERC1*, interferon regulatory factor 2-binding protein-like gene (*IRF2BPL*), histone-lysine N-methyltransferase 2D gene (*KMT2D*), leucine-rich repeat and sterile alpha motif containing one gene (*LRSAM1*), 43 kD receptor-associated protein of the synapse isoform 2 gene (*RAPSN*), Ring finger protein, LIM domain interacting gene (*RLIM*), and ring finger protein 216 gene (*RNF216*), which expands the spectrum of susceptible genes for PD (Table 4 [157], [158], [159], [160], [161], [162], [163], [164], [165], [166], [167], [168]).

**Table 4:** Rare variants of E3 ligases from the early onset PD population.

**Variants**	**Functions**	**Involved diseases**	**Note**	**References**
*HERC1*	Membrane trafficking, inflammation, neuromuscular transmission, spermatogenesis, iron metabolism, etc.	LRRK2 mutated PD, Angelman-like syndrome, *rjsf/df2* syndrome, cervical carcinogenesis	*HERC2* highly expressed in brain	[157], [158], [159]
*IRF2BPL*	Neuronal development and maintenance, Wnt signaling	NEDAMSS (neurodevelopmental disorder with regression, abnormal movements, loss of speech, and seizures), progressive myoclonus epilepsies	-	[160], [161], [162], [163]
*RAPSN*	Survival for motor neurons (agrin signaling)	Congenital myasthenic syndrome	-	[164]
*RNF216*	Αccumulation and spreading of phosphorylated tau, hypothalamic-pituitary-gonadal axis regulation	Bipolar disorder, autosomal recessive Huntington-like disorder	Also called TRIAD3	[165], [166], [167], [168]

“-” indicate no data. TRIAD3. TRAF interacting protein with a forkhead-associated domain and E3 ligase activity; HERC1. HECT and RLD domain containing E3 ubiquitin protein ligase family member 1; LRRK2. Leucine-rich repeat kinase 2; PD. Parkinson’s disease; IRF2BPL. Interferon regulatory factor 2 binding protein like; RAPSN. Receptor-associated protein of synapse; RNF216. Ring finger protein 216

HERC1 is involved in the maintenance and homeostasis of synaptic vesicles in motor nerve terminals. Targeting *HERC1* and its role in autophagy could offer therapeutic potential for neurological disorders linked to *HERC1* mutations, such as motor neuron diseases, by enhancing synaptic function and mitigating neurodegeneration [169]. In the context of neurodevelopmental disorders, a report detailed the pathological description of IRF2BPL-related disorder (NEDAMSS), characterized by neurodegeneration with regression, abnormal movements, loss of speech, and seizures. The patient exhibited pathological changes consistent with DRPLA, including polyQ inclusions, but genetic testing for DRPLA was negative. Subsequent exome sequencing revealed a pathogenic *de novo IRF2BPL* variant (c.562 C>T, p.Arg188Ter), which destabilizes the protein and disrupts Wnt signaling [170]. Turning to immune-autophagy crosstalk, RNF216 maintains cellular homeostasis by suppressing autophagy through BECN1 degradation and dampening TLR-mediated inflammation via ubiquitination of key signaling components (TLR4, TLR9, RIPK1, TRAF3); Dysregulated overexpression disrupts this balance, correlating with advanced disease stages and highlighting its potential as both a biomarker and therapeutic target in neurodegenerative disorders [171], [172].

#### 4.5.7 E3 ligase dysfunction across the neurodegenerative spectrum

A comparative analysis shows that, while E3 ligase dysfunction causes proteostasis failure in PD, AD, HD, and ALS, the mechanisms underlying this dysfunction differ across these diseases. These contrasting modalities, mitochondrial vs*.* synaptic targeting, K63 trapping vs*.* K48 degradation, compensatory upregulation vs*.* primary loss, and species- or cell-type-restricted activity, support the idea of disease-specific modulation of E3 networks rather than broad interference with ubiquitination. However, the recurring issues in the field include: mitophagy arrest, oligomer ubiquitination, synaptic UBE3A blockade, K63-mediated trapping, and ERAD collapse, all intensified by cross-seeding of tau, Aβ, and α-syn [64], along with an age-related pathological spectrum [9], and widespread mixed proteinopathies [3], which collectively depict neurodegeneration as a proteostasis continuum rather than as distinct disorders.

### 4.6 Other neurodegenerative diseases

#### 4.6.1 Dentatorubral-pallidoluysian atrophy

DRPLA is an autosomal-dominant ND caused by CAG repeat expansions in the *ATN1* gene, encoding a mutant atrophin-1 protein with an expanded polyQ tract. This mutant protein, along with its cleavage fragments, accumulates primarily in the nucleus, forming intranuclear inclusions. However, caspase-3 cleavage generates N-terminal polyQ-containing fragments that localize to both the cytoplasm and nucleus and readily form aggregates upon overexpression [173]. Notably, cells harboring these aggregates subsequently display apoptotic markers, suggesting that caspase-3-mediated fragmentation of mutant atrophin-1 contributes to neurodegeneration [145].

#### 4.6.2 Angelman syndrome

AS is an autosomal recessive neurodegenerative disorder that causes intellectual disability, seizures, lack of speech, and ataxia. Missense and nonsense point mutations in the *UBE3A* gene, which encodes the E3 ligase E6AP, lead to the inactivity of E6AP and are thought to contribute to AS by increasing aggregation of the mutant E6AP’s C-terminal lobe [174].

### 4.7 Remaining challenges

#### 4.7.1 Function-dependence of CHIP

While CHIP is generally viewed as neuroprotective, its mechanism varies depending on the context, hindering straightforward therapeutic translation. In models of PD and AD, CHIP aids in breaking down toxic α-syn oligomers and BACE1, respectively; in contrast, in HD models, CHIP reduces mHtt aggregation by promoting HSP70-dependent refolding rather than proteasomal degradation [114], [115], [119], [175], [176], [177]. In ALS, CHIP does not directly ubiquitinate mutant SOD1; instead, it targets the HSP70-SOD1 complex. These different mechanisms, degradation vs*.* refolding, direct vs*.* indirect ubiquitination, raise an important question. Does CHIP’s protective ability depend on the shape of aggregates, the cell’s stress environment, or levels of cell-specific chaperones? Additionally, CHIP overexpression can deplete HSP70, potentially impairing basal proteostasis and narrowing the therapeutic window [155], a scenario where neuroprotection may paradoxically become detrimental under acute stress.

#### 4.7.2 Causation vs*.* compensation

Many studies show that knocking down E3 ligase worsens the disease, while overexpression improves it, but this does not prove causality [178]. Reduced expression of Parkin, FBXO2, or MARCHF5 in diseased tissue could either be a primary cause of neurodegeneration or a secondary effect of a failing compensatory response overwhelmed by chronic proteotoxic stress. For example, the fact that CHIP “compensates for Parkin loss” in PD models suggests that upregulation might be a failed protective response rather than an initial defect [114]. To distinguish these scenarios, it’s necessary to conduct temporal studies that track ligase expression throughout disease progression and use conditional knockout models that maintain normal expression patterns [114], [137].

#### 4.7.3 Species and cell-type specificity

The identification of TRIM37 as a primate-specific E3 ligase for Htt, with selective vulnerability in the striatum, emphasizes that findings from rodent models may not always translate to human disease [144]. Similarly, SIAH-1-mediated mono- or di-ubiquitination of α-syn promotes aggregation specifically in oligodendrocytes in MSA, but the mechanisms underlying this cell-type specificity remain unclear [147]. Most functional studies rely on homogenized brain tissue or heterologous cell lines, which obscure the region-specific and cell-autonomous roles of E3 ligases across neuronal and glial populations [179], [180].

## 5 Therapeutic strategies targeting the E3 ligase for neurodegenerative diseases

**Fig. 4:**
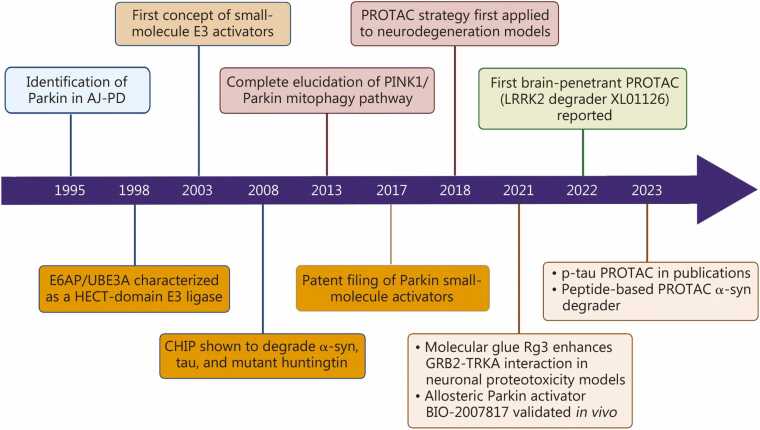
Development timeline of E3 ligase-directed therapies for neurodegenerative diseases. Key milestones span from the identification of *PRKN* mutations to the studies of α-syn PROTACs. AJ-PD. Autosomal juvenile Parkinson’s disease; PINK1. PTEN-induced putative kinase 1; E6AP/UBE3A. E6-associated protein/ubiquitin-protein ligase E3A; CHIP. C terminus of HSC70-interacting protein; GRB2. Growth factor receptor-bound protein 2; TRKA. Tropomyosin receptor kinase A; PROTAC. Proteolysis targeting chimera; α-syn. α-synuclein; LRRK2. Leucine-rich repeat kinase 2; PRKN. Parkin gene.

While the therapeutic potential of E3 ligases has been extensively explored in cancer treatment [181], their efficacy in NDs remains largely unknown. Strategies to make E3 ligases druggable include the use of E3 ligase activators and target protein degradation (TPD) technologies, such as proteolysis-targeting chimeras (PROTACs) [182] and molecular glues (Fig. 4).

### 5.1 E3 ligase activators

These activators can increase E3 ligase activity by inducing favorable allosteric changes [182]. When small molecules bind allosterically to E3 ligases, they modify the substrate-binding site, changing substrate specificity and thus influencing ubiquitination and protein degradation. A proline- and arginine-rich peptide, PR11, was modified by adding HbXY to its C-terminus, converting this allosteric 20S proteasome inhibitor into a potent proteasome activator. This engineered peptide promoted 20S proteasome-mediated degradation of substrates, including α-syn, and induced proteasome activation in fibroblasts [183]. Beyond enhancing proteasomal clearance, therapeutic strategies have also targeted the PINK1/Parkin mitophagy pathway. Small-molecule approaches include BIO-2007817, a tetrahydropyrazolo pyrazine (THPP) derivative that binds allosterically to autoinhibited Parkin and enhances the accessibility of the RING2 domain for Ub conjugation [184]. Alternatively, genetic engineering offers a complementary strategy: structure-guided hyperactive Parkin mutants (W403A and F146A) destabilize the REP interfaces between RING1 and RING0:RING2, thereby restoring mitophagy activity relative to pathogenic variants by facilitating conformational changes during PINK1-mediated activation [185].

### 5.2 Targeted protein degradation technologies

TPD technologies utilize the cell’s natural disposal systems to remove disease-causing proteins. This includes PROTACs and molecular glues that target the UPS [186], as well as various technologies that exploit the autophagy-lysosomal pathway. These autophagy-based methods include autophagosome-tethering compounds (ATTECs), which link targets to LC3; autophagy-targeting chimeras (AUTACs), which use guanine-derived tags for degradation; the autophagy-targeting chimera (AUTOTAC) platform [187], which directly activates p62/sequestosome 1 (SQSTM1) in a Ub-independent manner for degrading pathological aggregates; and lysosome-targeting chimeras (LYTACs), which target extracellular and membrane proteins. Together, these innovative approaches offer powerful ways to target traditionally “undruggable” proteins, such as transcription factors, non-enzymatic scaffolds, and aggregated proteins, by inducing their specific degradation [188], [189].

PROTACs are innovative bifunctional molecules designed to selectively degrade specific intracellular proteins by targeting them for degradation via the UPS. Each PROTAC consists of three components: a ligand for the protein of interest (POI), a ligand for an E3 Ub ligase, and a linker connecting the two [190]. This structure facilitates proximity-induced ubiquitination of the POI, leading to its subsequent proteasomal degradation. A key advantage of PROTACs lies in their catalytic mode of action: a single molecule can degrade multiple copies of a POI, thereby enabling greater efficiency and targeting “undruggable” proteins resistant to conventional small-molecule inhibitors or antibodies [190].

In cancer therapy, PROTACs have shown significant promise by degrading oncogenic drivers, such as kinases, epigenetic modulators, and anti-apoptotic proteins, potentially enabling lower therapeutic doses and less frequent administration [191]. Despite these advantages, PROTACs face formulation barriers: molecular weights (typically 700–1100 Da) exceed Lipinski’s rule [192], while high topological polar surface area (topological polar surface area >150 Å^2^) and lipophilicity (clogP 4–7) limit passive diffusion across the blood-brain barrier (BBB) [192]. Consequently, brain-to-plasma exposure ratios (Kp, uu) often fall below 0.1, as seen in tau-PROTACs (e.g., Kp, uu=0.03). Strategies such as prodrug masking of polar groups, nanoparticle encapsulation, or intracerebroventricular delivery are under investigation to enhance CNS bioavailability [193].

The clinical development of PROTACs is exemplified by vepdegestrant (ARV-471), the first PROTAC submitted to the Food and Drug Administration (FDA) for approval as an ER^+^ breast cancer treatment [191]. Despite its demonstrated effectiveness, ARV-471’s oral bioavailability is limited by intestinal enzymatic degradation, poor permeability, and P-gp-mediated efflux [194], [195]. These challenges highlight the need for robust optimization strategies, including prodrug design and advanced formulation approaches.

For next-generation methods, AbTACs (antibody-based PROTACs) provide a promising approach to targeting cell-surface proteins [196]. Important factors for optimization include selecting the appropriate E3 ligase [such as zinc and ring finger 3 (ZNRF3)], epitope mapping, and engineering the antibody scaffold to enhance ternary complex stability and degradation efficiency [197]. For traditionally undruggable targets like RAS, PROTAC development encounters specific challenges: 1) designing ligands for cryptic pockets (like Switch II), 2) optimizing linkers for effective ternary complex formation, 3) expanding the E3 ligase repertoire beyond traditional CRBN/von Hippel-Lindau (VHL), and 4) reducing resistance by targeting multiple mutants and inhibiting downstream pathways [198]. Other obstacles include GTP affinity, specificity for mutant vs*.* wild-type RAS, and suboptimal ADME (Absorption, Distribution, Metabolism, Excretion) properties of the drug. Addressing these issues involves iterative, structure-based design, combined with AI-driven linker prediction and peptide-based PROTACs that incorporate high-affinity biologics [198], [199].

Overall, a multidisciplinary optimization framework, combining empirical screening, computational modeling, and advanced synthetic chemistry, remains crucial for improving PROTAC stability, permeability, and therapeutic index [199]. Future efforts aim to expand the E3 ligase toolbox [200], develop new delivery methods such as nanoparticle-based PROTACs [201], and explore alternative degradation pathways to boost efficacy and versatility. Although many PROTACs targeting cancer are currently in clinical trials, their use in NDs is still in the preclinical stage. However, *in vitro* and animal studies have shown strong effectiveness against key ND-related substrates, including tau, GSK-3β, LRRK2, and α-syn, making PROTACs a promising new approach for ND therapy [192], [202], [203], [204], [205].

### 5.3 Molecular glues and genetic therapy

Beyond bifunctional degraders like PROTACs, alternative strategies to manipulate the proteostasis network are emerging. Molecular glues and genetic therapies targeting regulatory RNAs offer complementary mechanisms to modulate protein levels and interactions in NDs [206], [207], [208].

Molecular glues are small molecules that facilitate *de novo* protein-protein interactions by binding to one partner and creating a new interaction surface for the other [206]. While their application in NDs is nascent, they hold conceptual promise for redirecting pathogenic proteins toward degradation or inactivation. A relevant proof-of-concept is the ginsenoside Rg3 [209], which acts as a molecular glue to enhance the interaction between growth factor receptor-bound protein 2 (Grb2) and tropomyosin receptor kinase A (TrkA). This induced protein-protein interaction mitigates proteotoxicity *in vivo* [210], demonstrating that small-molecule-induced complex formation can ameliorate disease phenotypes. Although this example does not directly harness the UPS, it validates the principle of pharmacologically reprogramming protein interactomes, a strategy that could be adapted to recruit E3 ligases to pathogenic substrates.

MicroRNAs (miRNAs) play a crucial role in regulating the protein degradation machinery, and their dysregulation is linked to ND pathogenesis [207], [208]. Targeting specific miRNAs offers a genetic therapeutic approach to restore proteostasis by modulating E3 ligase activity. miR-9 is upregulated in AD and directly inhibits UBE4B and STUB1, E3 ligases that function synergistically to clear monomeric and oligomeric tau via ubiquitination-dependent autophagy [211]. This suppression compromises tau proteostasis, accelerating aggregation and disease progression. Inhibiting miR-9 activity would undo this suppression, allowing UBE4B and STUB1 to enhance E3 ligase-mediated degradation of tau aggregates.

### 5.4 Lessons from clinical oncology

Clinical trials of PROTACs have uncovered significant neurotoxicity risks that could lead to NDs. The mouse double minute 2 (MDM2) homolog activator Idasanutlin [212] caused grade 3 peripheral neuropathy and cognitive impairment in 12% of patients (NCT03107780), possibly related to p53-mediated neuronal stress or apoptosis, although the exact molecular mechanism remains unknown. Meanwhile, CRBN-based PROTACs (e.g., ARV-471) triggered lymphopenia and cytokine release syndrome by depleting IKAROS family zinc finger 1/3 (IKZF1/3), potentially hindering microglial Aβ clearance in AD [181], [213]. NX-2127 demonstrated a dangerously narrow therapeutic index (<3), with hepatotoxicity occurring at only three times the effective dose, warning that sustained CHIP activation could deplete HSP70 and render neurons vulnerable to acute stress [155], [214]. Additionally, the FDA (2024) PROTAC guidelines indicate that 77% of candidates fail to penetrate the CNS due to P-gp/BCRP efflux (Kp, uu<0.1) and hepatic CYP3A4-mediated linker oxidation, while off-target degradation of DNA repair proteins (e.g., BRCA1) raises long-term concerns about genomic instability [199].

These failures call for a shift in the ND therapeutics approach. First, for PD-specific patient stratification, focus on PRKN-null PD subpopulations, where E3 restoration provides the greatest benefit relative to risk. Second, dose selection requires real-time CNS biomarkers such as cerebrospinal fluid p-S65-Ub for Parkin activity and neurofilament light chain for neurodegeneration, to avoid the narrow therapeutic window trap of under- or over-activation and ensure therapeutic efficacy without compromising neuronal survival [215]. Third, engineering solutions include: 1) TRIM32-recruiting PROTACs that leverage neuron-specific E3 expression to limit degradation to the CNS; 2) prodrug strategies (tert-butyl masking) achieving Kp, uu>0.35 and MW<800 Da; and 3) reversible or opto-PROTACs to bypass feedback inhibition [216]. Importantly, investigational new drug (IND)-enabling studies must now incorporate human ether-à-go-go-related gene (hERG) cardiotoxicity and demyelination endpoints per the FDA 2024 guidance, recognizing that CNS-restricted delivery methods (such as Angiopep-2 nanoparticles or intracerebral infusion) may be necessary rather than optional for ND-focused degraders [217].

### 5.5 Challenges for neurologic translation

PROTAC development for NDs faces some challenges [218], [219], [220], while a recent BioPROTAC study for SOD1-associated ALS offers a clever solution: researchers used neuronal transgenic expression of an scFv-CHIP fusion, bypassing both delivery barriers and CRBN-related toxicity, successfully delaying disease progression and reducing misfolded aggregates in mice [221]. This innovative approach demonstrates the efficacy of TPD-based approaches. In spinal and bulbar muscular atrophy (SBMA), a polyQ expansion disorder, androgen receptor (AR)-targeting PROTACs recruit CRBN and bind the ligand-binding domain, succeed in prostate cancer trials, but fail to cross the BBB [222]. Alternative selective androgen receptor degraders (SARDs) that target the AR N-terminus avoid competition with endogenous ligands but face similar delivery challenges. Although muscle-targeted strategies rescue disease phenotypes in SBMA mouse models, achieving full therapeutic benefit requires both muscular and neuronal targeting to address neuromuscular issues [223], [224]. The ALS study underscores the urgent need for dual-optimized designs that balance brain penetration and molecular specificity, potentially using gene therapy vectors rather than traditional small molecules to comprehensively treat polyQ diseases.

### 5.6 Advances and trends in PROTAC

The evolution of PROTAC technology has progressed from initially recruiting 4 main E3 Ub ligases: CRBN [225], VHL [226], inhibitor of apoptosis protein (IAP), and MDM2, to more advanced, non-traditional designs that extend beyond conventional target limits [227]. While early PROTACs transformed intracellular protein degradation via the UPS, their limited ability to target cytosolic proteins necessitated alternative strategies for membrane- and extracellular proteins [228]. Emerging emPROTACs have addressed this challenge by integrating degradation principles with the endocytic trafficking machinery, exemplified by LYTACs, which utilize cell-surface receptors for lysosomal routing [229]. These spatially unrestricted platforms, including antibody-based PROTACs [230] and aptamer conjugates [231], have significantly expanded the target space. The latest innovation combines molecular glue stabilization with the PROTAC architecture, yielding MGPROTACs that hijack glues, such as fusicoccin, to stabilize protein-protein interfaces between disordered targets and scaffold proteins [232]. This approach allows cooperative recruitment of VHL E3 ligase to previously undruggable complexes. Confirmed by cryo-EM, which reveals new E3-scaffold interfaces, this hybrid method demonstrates that tunable linker chemistry can regulate ubiquitination specificity and efficiency, broadening the druggable universe beyond traditional ligand pockets to include dynamic, partially disordered protein assemblies [233].

To address PROTAC’s inherent limitations, such as high molecular weight, which reduces BBB penetration, and the risk of systemic off-target degradation, we propose a three-tiered integration strategy that combines emerging advances from glioblastoma and neurodegeneration research with established platform technologies [234] and the genome-wide degrader library discovered through proteome-scale screens [235].

Molecular miniaturization addresses PROTACs’ poor BBB permeability. Size-compressed pro-PROTACs replace heterobifunctional structures with compact photocaged prodrugs, in which a light-sensitive mask temporarily inactivates the VHL ligand, reducing molecular weight while preserving on-demand activation. π-π stacking drives spontaneous nanoprecipitation into 50–200 nm particles that exploit the enhanced permeability and retention effect in AD pathology [201]. For direct CNS delivery bypassing the BBB, implantable double-layer microneedles deposit prodrug depots directly into hippocampal/cortical tissue: the outer hyaluronic acid layer releases acid-activatable particles, while the inner methacrylated reservoir generates O_2_ from local H_2_O_2_ via MnO_2_ nanodots to alleviate hypoxia and facilitate near-infrared-triggered uncaging [236]. Alternatively, biomimetic vesicles cloaked in glioma cell membranes leverage homotypic adhesion to cross the intact BBB and accumulate 5.4-fold more in diseased tissue than free degraders [237]. Finally, spatiotemporal precision is achieved through dual-trigger activation: endolysosomal acidification (pH 4.5–6.0) combined with near-infrared irradiation (700–1000 nm) releases active PROTAC via photochemical uncaging and pH-sensitive cleavage. The liberated degrader then engages endoTAC’s multivalent sorting machinery, potentially recruiting E3 ligase effectors such as FBXL12, FBXL15, and KLHDC2, to enhance ternary complex assembly and lysosomal degradation [238]. This integrated platform localizes therapeutic activity to diseased tissue while minimizing systemic exposure (For detailed therapeutic candidates, please refer to Table 5 [70], [71], [73], [111], [128], [131], [147], [155], [184], [185], [192], [202], [203], [204], [205], [207], [208], [209], [211], [221], [232], [233]).

**Table 5:** Therapeutic candidates targeting E3 ligases in NDs.

Candidates	Targets	Mechanism	Trial	Disease	Key findings	**References**
Direct E3 ligase modulators						
BIO-2007817 (THPP scaffold)	Parkin (PRKN)	Allosteric activation; binds autoinhibited Parkin to increase RING2 domain accessibility	Preclinical	PD	Parkin activation *in vitro*; small-molecule approach	[184]
Hyperactive Parkin mutants (W403A, F146A)	Parkin (PRKN)	Structure-guided mutations destabilize repressor element, enhancing PINK1-mediated activation	Discovery	PD	Rescues mitophagy activity; research tool	[185]
Topotecan	UBE3A/E6AP	Topoisomerase inhibitor that unsilences paternal *UBE3A* allele expression	Phase I/II	AS	Restores cognitive function in mouse models; clinical trials ongoing	[70]
Antisense oligonucleotides	UBE3A/E6AP	Gene therapy to unsilence neuronal paternal *UBE3A*	Preclinical	AS	Ameliorates cognitive deficits in mouse models	[71], [73]
HSP70 activators (YM-1, MKT-077)	CHIP/STUB1	Stabilize ADP-bound HSP70, enhancing CHIP-HSP70 interaction and substrate ubiquitination	Preclinical	PD, AD, ALS	Increases degradation of misfolded proteins; broad applicability	[155]
miR-9 inhibitors	CHIP/STUB1, UBE4B	Antagomir that derepresses *STUB1* and *UBE4B* mRNA, restoring E3 ligase levels	Discovery	AD	Reduces tau pathology in preclinical models	[211]
*TRIP12* knockdown/antisense	TRIP12 (HECT)	siRNA or ASOs reducing TRIP12 expression	Discovery	PD	Restores GCase activity and reduces α-syn aggregation *in vitro*	[128]
SIAH-1 inhibitors	SIAH-1 (RING)	Small-molecule inhibitors blocking atypical α-syn ubiquitination	Discovery	MSA	Prevent toxic α-syn aggregation in oligodendrocytes	[147]
TRIM32 inhibitors	TRIM32 (TRIM)	Block mitochondrial translocation and XIAP degradation	Discovery	PD	Prevent mitochondrial dysfunction and apoptosis	[131]
*TRIM31* gene therapy	TRIM31 (TRIM)	Viral vector-mediated overexpression to enhance VDAC1 degradation	Discovery	PD	Protects dopaminergic neurons in mouse models	[111]
PROTACs & Targeted degraders						
Tau-PROTACs (peptide-based)	CRBN or VHL	Bifunctional molecules linking tau to E3 ligase for proteasomal degradation	Preclinical	AD	Tau reduction *in vitro*; BBB penetration challenges	[192], [202]
LRRK2-PROTAC (XL01126)	CRBN	Potent, selective LRRK2 degradation (DC_50_ in nmol/L range)	Preclinical	PD	Oral bioavailability; BBB penetration challenges remain	[204]
α-syn-PROTACs	CRBN/VHL	Target α-syn monomers/oligomers for UPS degradation	Preclinical	PD, MSA, Dementia with Lewy bodies	Early design stage; formulation barriers	[205]
GSK-3β PROTACs	CRBN/VHL	Degrade GSK-3β kinase, reducing tau hyperphosphorylation	Preclinical	AD	Demonstrated efficacy in neuronal models	[203]
BioPROTAC (scFv-CHIP)	CHIP/STUB1	Neuronal transgenic expression of scFv-CHIP fusion protein degrades misfolded SOD1	Preclinical	ALS	Delays disease progression in mouse models; avoids delivery/toxicity issues	[221]
Molecular glues & Emerging strategies						
Ginsenoside Rg3	Molecular glue	Enhances Grb2-TrkA interaction, mitigating proteotoxicity	Discovery	General neuroprotection	Proof-of-concept *in vivo;* not UPS-based but validates glue principle	[209]
MGPROTAC (fusicoccin-based)	VHL	Stabilizes target-scaffold interface for cooperative E3 recruitment	Discovery	Undruggable targets	Enables degradation of disordered protein complexes; cryo-EM validated	[232], [233]
miRNA-based therapies	Various E3s	Target miRNAs that regulate E3 ligase expression (e.g., miR-9)	Preclinical	AD, PD	Restores endogenous protein clearance pathways; systemic delivery challenges	[207], [208]

BIO-2007817. Bioactive compound 2007817; THPP. Tetrahydropyrazolo-Pyrazine; RING. Really interesting new gene; PINK1. PTEN induced putative kinase 1; UBE3A/E6AP. Ubiquitin protein ligase E3A/E6-associated protein; AS. Angelman syndrome; HSP70. Heat shock protein 70; CHIP/STUB1. C-terminus of HSC70-interacting protein/STIP1 homology and U-box containing protein 1; PD. Parkinson’s disease; AD. Alzheimer’s disease; ALS. Amyotrophic lateral sclerosis; miR-9. MicroRNA-9; UBE4B. Ubiquitination factor E4B; TRIP12. Thyroid hormone receptor interactor 12; HECT. Homologous to the E6-AP carboxyl terminus; siRNA. Small interfering RNA; GCase. Glucocerebrosidase; α-syn. α-synuclein; SIAH-1. Seven in Absentia homolog 1; MSA. Multiple system atrophy; TRIM. Tripartite motif; XIAP. X-linked inhibitor of apoptosis protein; VDAC1. Voltage-dependent anion channel 1; PROTACs. Proteolysis-targeting chimeras; CRBN. Cereblon; VHL. von Hippel-Lindau; BBB. Blood-brain barrier; LRRK2. Leucine-rich repeat kinase 2; XL01126. Experimental compound XL01126; UPS. Ubiquitin-proteasome system; GSK-3β. Glycogen synthase kinase 3β; BioPROTAC. Biologic proteolysis-targeting chimera; scFv. Single-chain fragment variable; SOD1. Superoxide dismutase 1; ARV-471. Androgen receptor degrader-471; ND. Neurodegenerative disease; CNS. Central nervous system; MGPROTAC. Molecular glue proteolysis-targeting chimera; EM. Electron microscopy; Grb2. Growth factor receptor-bound protein 2; TrkA. Tropomyosin receptor kinase A

### 5.7 Conclusions and perspective

E3 ligases are central to proteostasis failure, the shared pathology of all neurodegenerative diseases. While their therapeutic exploitation faces challenges, such as multisubstrate promiscuity, context-dependent mechanisms, and delivery barriers, emerging strategies offer viable solutions. PROTACs and molecular glues now expand beyond traditional E3s (CRBN, VHL) to neuron-specific ligases and undruggable targets; miRNA-based approaches restore endogenous degradation capacity; and engineered E3 variants (hyperactive Parkin, scFv-CHIP) circumvent off-target toxicity. Translation to the clinic requires addressing three critical gaps: achieving CNS penetration without systemic exposure, distinguishing causative dysfunction from compensatory responses, and validating efficacy in human-relevant models. Success in these areas will establish E3-directed therapies as a foundational strategy for neurodegeneration, moving from symptomatic management to disease-modifying intervention.

## Abbreviations

α-syn: α-synuclein

Aβ: Amyloid-β

AD: Alzheimer’s disease

ALS: Amyotrophic lateral sclerosis

APP: Amyloid precursor protein

AR: Androgen receptor

AS: Angelman syndrome

ASO: Antisense oligonucleotide

ATN1: Atrophin-1

AUTAC: Autophagy-targeting chimera

AUTOTAC: Autophagy-targeting chimera (platform)

BACE1: β-site APP-cleaving enzyme 1

BBB: Blood-brain barrier

CCNF: Cyclin F

CHIP: C-terminus of HSC70-interacting protein (gene name *STUB1*)

CK2α: Casein kinase 2α

C-lobe: C-terminal lobe

CRBN: Cereblon

DRPLA: Dentatorubral-pallidoluysian atrophy

E6AP: E6-associated protein

ER: Endoplasmic reticulum

ERAD: ER-associated degradation

FBXO2: F-box only protein 2

FBXW7: F-box and WD repeat domain containing 7

FTD: Frontotemporal dementia

GCase: Glucosylceramidase

GCI: glial cytoplasmic inclusion

GSK-3β: Glycogen synthase kinase 3β

HD: Huntington’s disease

HECT: Homologous to E6AP C-terminus

HERC: HECT and RCC1-like domain containing

hERG: Human ether-à-go-go-related gene

HRD1: HMG-CoA reductase degradation protein 1

HSP70: Heat-shock protein 70

HSF1: Heat shock transcription factor 1

Htt: Huntingtin

IAP: Inhibitor of apoptosis protein

IC_50_: Half-maximal inhibitory concentration

iNOS: Inducible nitric-oxide synthase

iPSC: Induced pluripotent stem cell

IRF2BPL: Interferon regulatory factor 2 binding protein-like

K_i_: Inhibition constant

LC3: Microtubule-associated protein 1 A/1B-light chain 3

LRRK2: Leucine-rich repeat kinase 2

LYTAC: Lysosome-targeting chimera

m^6^A: N^6^-methyladenosine

MARCH: Membrane-associated RING-CH

MASLD: Metabolic dysfunction-associated steatotic liver disease

MDM2: Mouse double minute 2

SNpc: Substantia nigra pars compacta

SOD1: Superoxide dismutase 1

mHtt: Mutant huntingtin

MSA: Multiple system atrophy

NDs: Neurodegenerative diseases

Nedd4: The neural precursor cell expressed developmentally down-regulated protein 4

NEDL1: Nedd4-like E3 ligase 1

NEDAMSS: Neurodevelopmental disorder with regression, abnormal movements, loss of speech, and seizures

NF-κB: Nuclear factor κB

N-lobe: N-terminal lobe

ORAI1: ORAI calcium release-activated calcium modulator 1

PA-TM-RING: Protease-associated transmembrane RING

PD: Parkinson’s disease

PINK1: Phosphatase and tensin homolog-induced kinase 1

PIAS1: Protein inhibitor of activated STAT 1

POI: Protein of interest

PP2A: Protein phosphatase 2A

PRKN: Parkin gene

PROTAC: Proteolysis-targeting chimera

RapSN: 43 kD receptor-associated protein of the synapse

RBR: RING-between-RING

RCC1: Regulator of chromosome condensation 1

REP: Repressor element protein

RING: Really interesting new gene

RIPK1: Receptor-interacting protein kinase 1

RNF220: Ring finger protein 220

ROS: Reactive oxygen species

SBMA: Spinal and bulbar muscular atrophy

SCF: Skp1-Cullin1-F-box protein

SIAH-1: Seven in absentia homolog 1

Smurf1: SMAD-specific ubiquitin regulatory factor 1

STUB1: STIP1 homology and U-box containing protein 1

SUMO: Small ubiquitin-like modifier

SYVN1: Synoviolin 1

TDP-43: TAR DNA-binding protein 43

TF: Transcription factor

TFEB: Transcription factor EB

THPP: Tetrahydropyrazolo-pyrazine

TLR4: Toll-like receptor 4

TPD: Target protein degradation

TRAF6: Tumor necrosis factor receptor-associated factor 6

TRIM: Tripartite motif

TRIM31: Tripartite motif-containing protein 31

TRIP12: Thyroid hormone receptor-interacting protein 12

Ub: Ubiquitin

UBE3A: Ubiquitin protein ligase E3A

UPS: Ubiquitin-proteasome system

VDAC1: Voltage-dependent anion channel 1

VHL: Von Hippel-Lindau

WWP1: WW domain-containing E3 ligase 1

XIAP: X-linked inhibitor of apoptosis protein

ZNRF3: Zinc and ring finger 3

## Ethics approval and consent to participate

Not applicable.

## Funding

This work was supported by the Singapore National Medical Research Council (NMRC) grants, including the Open Fund Large Collaborative Grant (MOH-OFLCG24may-0004) and the Singapore Translational Research (STaR) Investigator Award (NMRC/STaR/0030/2018), the Open Fund-Individual Research Grant (OF-IRG, MOH-001506), the Clinician Scientist-Individual Research Grants (CS-IRG) (MOH-001091, CIRG23jul-0006), the Human Longevity Catalysis Award 2024 (HLCA24Mar-0019), the Singhealth-Duke-NUS AM Position grant, and the National Natural Science Foundation of China (82171243).

## Data Availability

Not applicable.
